# Disease progression and search for monogenic diabetes among children with new onset type 1 diabetes negative for ICA, GAD- and IA-2 Antibodies

**DOI:** 10.1186/1472-6823-10-16

**Published:** 2010-09-23

**Authors:** Sven Pörksen, Lene Bjerke Laborie, Lotte Nielsen, Marie Louise Max Andersen, Tone Sandal, Heidi de Wet, Erik Schwarcz, Jan Åman, Peter Swift, Mirjana Kocova, Eugen J Schönle, Carine de Beaufort, Philip Hougaard, Frances Ashcroft, Anders Molven, Mikael Knip, Henrik B Mortensen, Lars Hansen, Pål R Njølstad

**Affiliations:** 1 Department of Pediatrics, Glostrup Hospital & University of Copenhagen, Copenhagen, Denmark; 2 Department of Clinical Medicine, University of Bergen, Bergen, Norway; 3 Department of Pediatrics, Haukeland University Hospital, Bergen, Norway; 4 Center for Medical Genetics and Molecular Medicine, Haukeland University Hospital, Bergen, Norway; 5 Gade Institute, University of Bergen, Bergen, Norway; 6 Department of Physiology, Anatomy and Genetics, University of Oxford, Oxford, UK; 7 Department of Pediatrics, University Hospital Ørebro, Ørebro, Sweden; 8 Department of Pediatrics, Leicester Royal Infirmery Children's Hospital, Leicester, UK; 9 Department of Endocrinology and Genetics, Paediatric Clinic, Skopje, Former Yugoslav Republic of Macedonia; 10 Department of Pediatrics, University Childrens Hospital, Zurich, Switzerland; 11 Clinique Pédiatrique, Centre Hospitalier de Luxembourg, Luxembourg; 12 Department of Biostatistics, University of Southern Denmark, Odense, Denmark; 13 Department of Pediatrics, Hospital for Children and Adolescents, University of Helsinki, Helsinki, Finland

## Abstract

**Background:**

To investigate disease progression the first 12 months after diagnosis in children with type 1 diabetes negative (AAB negative) for pancreatic autoantibodies [islet cell autoantibodies(ICA), glutamic acid decarboxylase antibodies (GADA) and insulinoma-associated antigen-2 antibodies (IA-2A)]. Furthermore the study aimed at determining whether mutations in *KCNJ11*, *ABCC8*, *HNF1A, HNF4A *or *INS *are common in AAB negative diabetes.

**Materials and methods:**

In 261 newly diagnosed children with type 1 diabetes, we measured residual β-cell function, ICA, GADA, and IA-2A at 1, 6 and 12 months after diagnosis. The genes *KCNJ11, ABCC8*, *HNF1A, HNF4A *and *INS *were sequenced in subjects AAB negative at diagnosis. We expressed recombinant K-ATP channels in Xenopus oocytes to analyse the functional effects of an ABCC8 mutation.

**Results:**

Twenty-four patients (9.1%) tested AAB negative after one month. Patients, who were AAB-negative throughout the 12-month period, had higher residual β-cell function (*P *= 0.002), lower blood glucose (*P *= 0.004), received less insulin (*P *= 0.05) and had lower HbA_1c _(*P *= 0.02) 12 months after diagnosis. One patient had a heterozygous mutation leading to the substitution of arginine at residue 1530 of SUR1 (*ABCC8) *by cysteine. Functional analyses of recombinant K-ATP channels showed that R1530C markedly reduced the sensitivity of the K-ATP channel to inhibition by MgATP. Morover, the channel was highly sensitive to sulphonylureas. However, there was no effect of sulfonylurea treatment after four weeks on 1.0-1.2 mg/kg/24 h glibenclamide.

**Conclusion:**

GAD, IA-2A, and ICA negative children with new onset type 1 diabetes have slower disease progression as assessed by residual beta-cell function and improved glycemic control 12 months after diagnosis. One out of 24 had a mutation in *ABCC8*, suggesting that screening of *ABCC8 *should be considered in patients with AAB negative type 1 diabetes.

## Background

Type 1 diabetes (T1D) is thought to result from an immune-mediated destruction of the pancreatic beta-cells in genetically susceptible people. The risk for developing T1 D seems to increase with genetic susceptibility in combination with the presence of immunological markers of beta-cell autoimmunity. Although the destruction of the pancreatic beta-cell is perceived to be mediated by T cells, the loss of immunological self-tolerance may result in autoantibody formation. Signs of immunological activity directed against the pancreatic beta-cell may appear many years before clinical disease presentation and predict the progression to type T1 D. Although not directly involved in beta-cell death, autoantibodies can be used as markers of beta-cell destruction and reflect disease severity. A subclass of T1 D children does not show any of these signs of humoral autoimmunity and are considered to have idiopathic or Type 1 B diabetes. Children and adolescents with newly diagnosed type 1 diabetes are more likely to present with several autoantibodies than adults, probably reflecting a stronger autoimmune state and a more severe disease progression.

Recently, there have been substantial improvements in molecular genetic diagnostics of diabetes in infants. A molecular diagnosis is now possible for glucokinase deficiency (1), mutations in transcription factors HNF-1α (2) or HNF-4 α (3), insulin gene mutations (4), and mutations in the pancreatic ATP-sensitive potassium (K-ATP) channel subunits KIR6.2 (5) and SUR1 (6, 7). Since oral treatment with sulfonylurea has become an attractive alternative for most of these patients, efforts should be made to diagnose these defects in patients with absence of autoantibodies against pancreatic antigens (8, 9, 10).

The aims of the present study were: 1) to compare the disease progression of type 1 diabetes among children negative and children positive for ICA, GADA and IA-2A, (subsequently referred to as autoantibody negative and autoantibody positive) during the first 12 months after disease onset, and 2) to investigate whether mutations in the *KCNJ11*, *ABCC8*, *HNF1A, HNF4A *or *INS *genes are common in children and adolescents with AAB negative diabetes.

## Methods

### Subjects

This is a multicenter longitudinal investigation with 18 participating pediatric centers from 15 countries in Europe and Japan. A total of 261 children and adolescents (132 girls, 129 boys, 84% Caucasian, 16% other ethnicities) up to 16 years of age were followed for 12 months from the diagnosis of T1D: Clinical information on demographics and anthropometry, insulin therapy as well as blood samples for centralized measurement of HbA_1c _and meal-stimulated C-peptide, proinsulin, and GLP-1 were collected prospectively. Exclusion criteria were: clinically suspected type 2 diabetes, diabetes in 3 consecutive generations with onset before age 25 (to exclude maturity onset diabetes of the young (MODY)), secondary diabetes, decline of enrolment into the study, and patients initially treated outside the centers for more than 5 days. Insulin regimens were recorded 1, 3, 6, 9 and 12 months after diagnosis. After 12 months, 52.9% of the children were on twice insulin daily. Only three children used an insulin infusion pump while 13% were treated with a rapid acting insulin analogue. Daily insulin dose was 0.72 ± 0.28 IU/kg (mean ± SD).

The study was performed according to the criteria of the Helsinki II Declaration and was approved by the local ethical committee in each centre. All patients, their parents or guardians gave written informed consent.

### Glycemic control

Glycemic control as assessed by HbA_1c _was measured at diagnosis, and at 1, 3, 6, 9 and 12 months after diagnosis. HbA_1c _was determined centrally by ion-exchange high-performance liquid chromatography (reference range 4.1-6.4%) at Steno Diabetes Center, Gentofte, Denmark. We used insulin dose adjusted **HbA1c (IDAA1c) = HbA1c (%) + [4 × insulin dose (U/Kg/24h)] **as a marker of disease severity. This measure, adjusting for the exogenous insulin, reflects the underlying and theoretically untreated disease, as it mimics a situation in which no insulin was administered. In this setting it therefore reflects the severity of the disease and hence is superior to the HbA_1c _alone (11).

### Antibodies

As all patients were treated with insulin during the first month after diagnosis, secondary insulin antibodies (IA) could not be distinguished from primary insulin autoantibodies (IAA) and, therefore, were not included in the classification of the antibody pattern at 1 month. Patients with detectable antibodies (IA-2A, GADA and/or ICA) at 1 month were considered autoantibody-positive. Patients with an absence of autoantibodies (IA-2A, GADA and/or ICA) at 1 month were considered autoantibody-negative.

ICA were detected by indirect immunofluorescence using commercial Primate Pancreas slides from INOVA. The sera were screened at a dilution of 1:2 and FITC-labelled anti-human IgG (Dako, Copenhagen, Denmark) was used as conjugate and grouped as negative 0-0.5 U.

GADA were quantified by a direct radioimmuassay (Diamyd Diagnostics, Stockholm, Sweden). Sera were run in duplicate, and the results were read on a gamma counter (Wizard 1470; Wallac/PerkinElmer, Turku, Finland) and calculated from a standard curve. The cut-off limit was 10 U/ml, set from a comparison between 94 patients with type 1 diabetes and 98 healthy blood donors. The intra- and interassay coefficients of variation were 2.9% and 5.1%, respectively (12).

IA-2A were analyzed with a radiobinding assay (13). The results were expressed as relative units (RU) based on a standard curve run on each plate using an automated calculation program (MultiCalc; Wallac). The limit for IA-2A positivity (0.77 RU) was set at the 99th percentile in 374 non-diabetic children and adolescents. The inter-assay coefficient of variation was < 12%. IA were measured by a modification of the method described by Williams et al. (14), the cut-off limit for positivity was 2.80 RU.

### Residual beta-cell function

Mixed-meal stimulated serum C-peptide was used as a marker of residual β-cell function after a disease duration of 1, 6, and 12 months. Serum samples were labeled and frozen at - 20 °C until shipment on dry ice for the determination of C-peptide within half a year.

Serum C-peptide was analyzed by a fluoroimmunometric assay (AutoDELFIATM C-peptide, Perkin Elmer Life and Analytical Sciences, Inc, Turku, Finland). The analytical sensitivity was better than 5 pmol/l, the intra-assay coefficient of variation was <6% at 20 pmol/l, and recovery of standard, added to plasma before extraction, about 100% when corrected for losses inherent in the plasma extraction procedure. Total proinsulin-immunoreactivity was analyzed by a two-site ELISA based on the monoclonal antibodies coating antibody PEP-001 and detecting antibody HUI-001 (Novo Nordisk A/S, Bagsværd, Denmark). The sensitivity was below 0.3 pmol/l.

### Genotyping

Typing of the HLA-class II *DRB1 *locus was performed by direct sequencing of exon 2 of *DRB1 *according to the Immuno Histocompatibility Working Group. *DR 03/04 *and *DR 04/04 *were defined as high-risk genotypes, while *DR 03/03 *and *DR 04/08 *were considered to convey moderate risk. All other genotypes were classified as low-risk. The analysis of the variable number of tandem repeats region of the insulin gene (*INS-VNTR*) was performed as previously described by Nielsen et al. (15) We sequenced coding exons and flanking intronic regions of the genes *HNF1A *(16), *HNF4A *(17)*, INS *(18) and *KCNJ11 *(5). All 39 exons, flanking intron and non-coding 5' and 3' untranslated regions of *ABCC8 *were sequenced by a high-throughput, semi-automated strategy as decribed by Sandal et al. (19).

### Functional analysis

For functional analysis, we coexpressed wild-type or mutant SUR1 together with wild-type Kir6.2 in *Xenopus laevis *oocytes, as previously described (6). Whole-cell currents were recorded using a 2-electrode voltage-clamp in response to voltage steps of ± 20 mV from a holding potential of -10 mV, in a solution containing (in mM): 90 KCl, 1 MgCl_2_, 1.8 CaCl_2_, 5 HEPES (pH 7.4 with KOH). Metabolic inhibition was induced by 3 mM Na-azide, and 0.5 mM tolbutamide was used to block K_ATP _channels, as indicated. Data were analysed with pCLAMP8 (Axon Instruments, CA, USA), Origin 6.02 (Microcal Software, Northampton, MA, USA) and Igor (Wavemetrics, Lake Oswego, OR, USA) software and are given as mean+SEM. Statistical significance was evaluated using an unpaired two-tailed Student's t-test. A probability value of P < 0.05 was considered a significant difference.

### Statistical methods

C-peptide, and proinsulin were investigated by means of the logarithmic scale. C-peptide, proinsulin, blood glucose change, insulin dose, HbA_1c _and IDAA1c were analysed by multiple regression with age, sex, and antibody status (negative/positive) as explanatory variables in a compound symmetric repeated measurement model. A *P*-value < 0.05 was considered significant. Statistical analyses were performed using SAS version 9.1 (SAS Institute, USA, Inc, Cary, NC, USA).

## Results

Twenty-four patients (9.1%, Table 1 and 2) out of 261 tested negative for GADA, IA-2A, and ICA after 1 month. Of these, 22 were also negative at 6 and 12 months after diagnosis, whereas one of the patients seroconverted to positivity at six months (IA-2A) and another at 12 months (GADA). The group of 22 patients remaining autoantibody-negative (Table1) did not differ significantly from the autoantibody-positive group with respect to sex (*P *= 0.40), age (*P *= 0.49), ethnicity (*P *= 0.80,), HLA risk groups (p = 0.68) or *INS-VNTR *genotypes. Autoantibody-negative patients had lower titers of Insulin Antibodies at 1 month after diagnosis (*P *= 0.01), not shown. Six of 22 (27%) autoantibody-negative patients had first-degree relatives with diabetes compared with 22 of 237 autoantibody-positive subjects (10%) (*P *= 0.01) indicating that genetic factors may be of predominant importance. None of the 6 autoantibody-negative patients with a family history of diabetes had mutations in *HNF1A *or *HNF4A*. There were no *INS *gene mutations in the autoantibody negative group.

**Table 1:** Clinical characteristics of the 22 study participants who remained negative for autoantibodies: GADA, IA-2A, and ICA 1, 6, and 12 months after diagnosis of type 1 diabetes

Patient	Age	Sex	BMI (kg/m^2^)	HbA1C 0 (%)	IDAA1C 1	IDAA1C 6	IDAA1C 12	Ins dose1 (U/kg/day)	ins dose6 (U/kg/day)	Ins dose12 (U/kg/day)	Cpep1 (pmol/L)	Cpep6 (pmol/L)	Cpep12 (pmol/L)	BGstim1 (mmol/L)	BGstim6 (mmol/L)	BGstim12 (mmol/L)
1	16.3	female	31.14	11.8	Na	7.1	6.8	0.33	0.22	0.33	1055	201	272	14.33	10	9.1

3	6.6	female	14.31	6.3	Na	Na	7.3	Na	Na	0.22	416	79	208	8.7	7.5	10.6

4	14.2	female	14.9	12.8	11.5	8.5	10	0.24	0.22	0.51	902	617	735	8.9	11.4	15.9

6	6.7	male	12.8	9.1	10.2	8.1	8.7	0.33	0.3	0.46	392	413	277	13.7	14.5	11.4

7	1.4	male	13.7	11.1	10.3	8.2	10.6	0.25	0.23	0.5	184	67	21	13.2	13.4	15.6

9	7	male	14.26	9	9.2	9.4	11.7	0.33	0.38	0.74	18	21	13	8.9	10.2	8.4

10	10.8	female	17.6	10.4	10.6	8.4	8.6	0.63	0.49	0.56	592	472	320	11.1	19.9	20.7

11	14.2	female	15.1	14.2	15.6	10.9	10.8	1.10	0.77	0.71	411	347	291	15.6	18.7	20.7

12	4.7	female	15.7	9.3	11.1	8.2	9.4	0.71	0.52	0.6	238	299	212	7.8	11.7	18.3

14	14.1	male	31.6	12	10.1	6.4	6.5	0.18	0.1	0.1	1353	1517	1147	6.2	5.8	5.0

15	3.6	male	11.5	15.3	13.2	9.8	13.7	0.44	0.39	0.84	479	380	216	10.1	Na	Na

16	11.2	male	16.4	10.9	11.7	7.1	8.0	0.63	0.2	0.41	577	294	590	10.3	7.2	9.2

17	3	female	17.72	8.3	8.6	7.4	7.1	0.39	0.3	0.57	379	252	2721	8.7	5.7	11.4

18	6.3	female	17.2	9.3	9.5	Na	7.8	0.48	0.41	0.38	417	886	495	9.4	12.3	11.7

19	11.3	female	14.4	13.2	12.5	10.8	11.1	0.70	0.94	0.89	514	376	437	14.7	10.7	12.7

20	14	male	19.4	13	12.5	7.4	11.3	0.52	0.37	0.44	374	272	615	10.3	8.9	16.3

21	3.1	male	14.4	11	9.9	9.0	Na	0.25	0.45	0.75	91	101	183	12.5	17.7	19.2

22	3.1	male	14.4	8.6	9.3	9.8	11.0	0.35	0.41	0.64	309	229	176	15.0	18.0	19.9

23	10.5	male	17.3	14.3	Na	11.7	13.0	0.91	1.0	1.4	312	210	156	10.4	14.4	15.7

25	11.1	female	Na	13	11.3	11.7	13.0	0.90	1.0	1.44	292	628	450	7.1	11.1	12.9

26	11.1	female	27.4	10.2	10.9	7.3	7.6	0.52	0.57	0.5	2040	1226	701	6.9	6.3	4.1

27	8.8	female	Na	9	9.4	8.5	10.1	0.30	0.31	0.69	596	482	318	9.4	23.6	20.3

Mean	8.4		16.9	11.0	10.9	8.9	9.9	0.49	0.47	0.64	518.4	436.6	489.6	10.4	12.5	14.0

SD	4.2		4.9	2.4	1.7	1.6	2.1	0.24	0.27	0.32	448.6	375.8	577.5	2.7	5.1	5.1

Abbreviations: SD, standard deviation; Na, not available; BMI, body mass index; HBA1C0, HBA1C at diagnosis; IDAA1C 1, Insulin Dose Adjusted HbA1c (IDAA1C) at 1 month; IDAA1C 6, IDAA1C at 6 months; IDAA1C 12, IDAA1C at 12 months; Ins dose1, insulin dose at 1 month; Ins dose6, insulin dose at 6 months; Ins dose12, insulin dose at 12 months; Cpep1, stimulated C-peptide at 1 month, Cpep6, stimulated C-peptide at 6 months; Cpep12, stimulated C-peptide at 12 months; BGstim1, 90 min glucose at 1 month; BGstim6, 90 min glucose at 6 months; BGstim12, 90 min glucose at 12 months

**Table 2:** Clinical characteristics of the two study participants who tested negative for autoantibodies (GADA, IA-2A, and ICA) at 1 month and who converted to positivity for GADA (patient 2) at 12 months or IA-2A (patient 24 (carrier of the Arg1530Cys mutation of the ABCC8)) at 6 and 12 months after the diagnosis of type 1 diabetes

Patient	Age (years)	Sex	BMI (kg/m^2^)	HbA1C0 (%)	IDAA1C 1	IDAA1C 6	IDAA1C 12	Ins dose1 (U/kg/day)	ins dose6 (U/kg/day)	Ins dose12 (U/kg/day)	Cpep1 (pmol/L)	Cpep6 (pmol/L)	Cpep12 (pmol/L)	BGstim1 (mmol/L)	BGstim6 (mmol/L)	BGstim12 (mmol/L)
2	16.3	female	17	10.9	Na	11.1	11.7	0.54	0.73	0.59	120	10	10	14.2	20.7	13.2

24	14	male	16.4	12.4	11.3	10.6	12.8	0.63	0.73	0.98	356	308	218	13.9	21	20.6

Abbreviations: Na, not available; BMI, body mass index; HBA1C0; HBA1C at diagnosis; IDAA1C 1, Insulin Dose Adjusted HbA1c (IDAA1C) at 1 month; IDAA1C 6, IDAA1C at 6 months; IDAA1C 12, IDAA1C at 12 months;; Ins dose1, insulin dose at 1 month; Ins dose6, insulin dose at 6 months; Ins dose12, insulin dose at 12 months; Cpep1, stimulated C-peptide at 1 month, Cpep6, stimulated C-peptide at 6 months; Cpep12, stimulated C-peptide at 12 months; BGstim1, 90 min glucose at 1 month; BGstim6, 90 min glucose at 6 months; BGstim12, 90 min glucose at 12 months;

### Better residual beta-cell function and glycemic control in autoantibody-negative subjects

**Figure 1:**
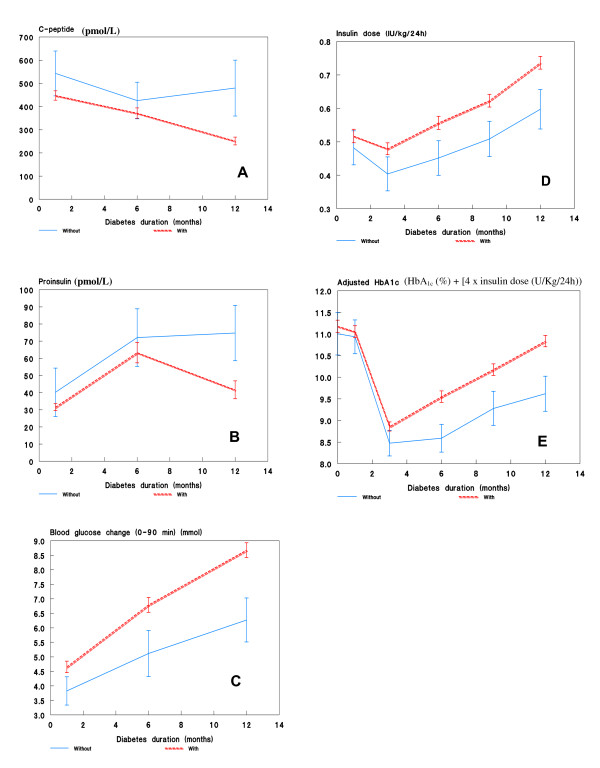
**Comparison of disease course in autoantibody-negative and autoantibody-positive children**: *A*: 12 months after disease onset, the residual beta cell function in autoantibody-negative patients was twofold higher than in autoantibody-positive patients (p = 0.002). *B*: Autoantibody-negative patients had significantly higher proinsulin release 12 months after disease diagnosis than autoantibody-positive subjects (*P *= 0.01). *C*: The blood glucose change (90 minutes value minus fasting value) during meal-stimulation differed significantly between autoantibody-negative and autoantibody-positive patients 12 months after disease onset (*P *= 0.004). *D*: 12 months after diagnosis autoantibody-negative patients received about 0.15 IU/kg/day less insulin than autoantibody-positive subjects (*P *= 0.02). *E*: Autoantibody-negative subjects had 0.8% lower IDAA1c than autoantibody-positive patients (*P *= 0.02).

The residual beta-cell function (as assessed by meal-stimulated C-peptide) in the 22 persistently autoantibody-negative patients was twice as high as in autoantibody-positive patients 12 months after diagnosis (*P *= 0.005, Fig. 1A), and the proinsulin values were correspondingly higher in autoantibody-negative patients (*P *= 0.01, Fig. 1B). Moreover, the autoantibody-negative patients experienced lower blood glucose changes (2.4 mmol/L) during the Boost test at 12 months of follow-up (*P *= 0.004, Fig. 1C). During the 12 months follow-up glycemic control was significantly better in autoantibody-negative patients as they had 0.65% (absolute) lower HbA_1c _than autoantibody-positive subjects (*P *= 0.04, data not shown). In terms of insulin requirement, the autoantibody-negative patients received 0.15 IU/kg/day less insulin 12 months after diagnosis compared with autoantibody-positive subjects (*P *= 0.02) (Fig. 1D). Twelve months after diagnosis, autoantibody-negative children had on an average 1.25% (absolute) lower insulin dose adjusted HbA1c (IDAA1c) than autoantibody-positive children (*P *= 0.005) (Fig. 1E).

### Mutation in *ABCC8*

We screened both the *KCNJ11 *and *ABCC8 *genes in all 24 subjects negative for autoantibodies (IA-2A, GADA and/or ICA) at one month after diagnosis. None had a mutation in *KCNJ11*. However, we identified a novel heterozygous mutation in *ABCC8*: a C > T change leading to a predicted Arg > Cys substitution at codon 1530 of SUR1 (Table 2) in one subject. This amino acid residue is conserved from zebrafish to humans and is located in the second nucleotide-binding domain of SUR1, a region previously implicated in neonatal diabetes.

**Figure 2:**
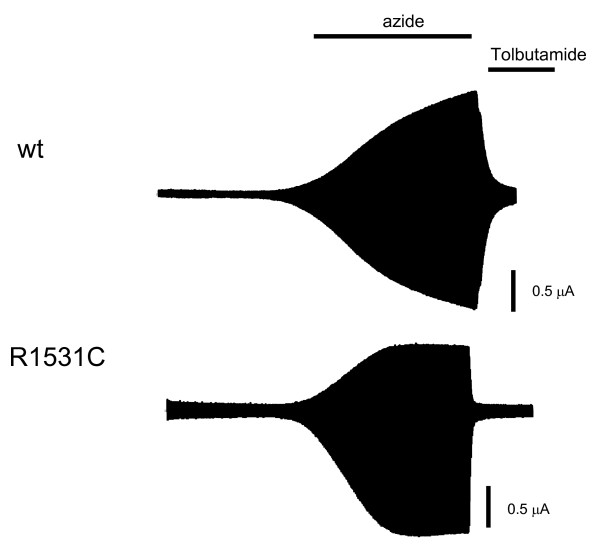
**Tolbutamide response in SUR1-R1530C**. Whole-cell currents recorded from Xenopus oocytes coexpressing Kir6.2 and either SUR1 (WT) or SUR1-R1530C in response to voltage steps of +20 mV from a holding potential of -10 mV. Bars indicate the times of application of 3 mmol/l azide or 0.5 mmol/l tolbutamide.

**Figure 3:**
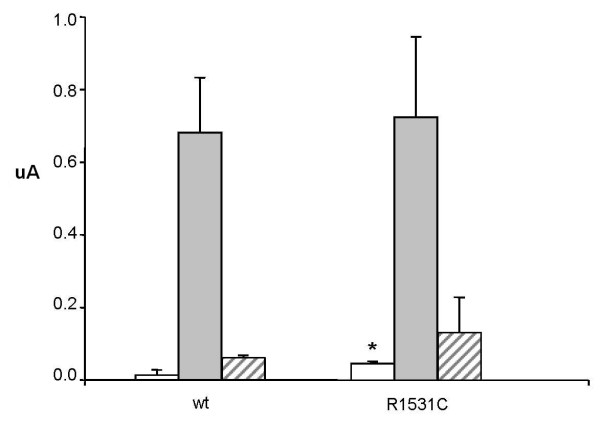
**Tolbutamide response in SUR1-R1530C**. Mean steady-state whole-cell K-ATP currents (as indicated) evoked by a voltage step from -10 to -30 mV before (rest; grey bars) and after application of 3 mmol/l azide (grey bars) and in the presence of 3 mmol/l azide plus 0.5 mmol/l tolbutamide (black bars). Four oocytes were used for each experiment. *P < 0.05against control (t-test).

We studied the functional effects of the SUR1-R1530C mutation by expressing recombinant K_ATP _channels in *Xenopus *oocytes. Wild-type K_ATP _channels are normally closed when expressed in *Xenopus *oocytes due to the high intracellular ATP concentration, but they are activated by metabolic inhibitors such as azide, which lower ATP (Fig. 2). In contrast, in oocytes expressing SUR1-R1530C mutant channels significant resting whole-cell K_ATP _currents were present in the absence of metabolic inhibition (Fig. 2). Thus, basal cellular metabolism causes less block of mutant K_ATP _channels than wild-type channels. Mutant channel currents were increased by 3 mmol/l azide, indicating that they can be further activated by metabolic inhibition. Importantly, mutant channels were blocked by 0.5 mmol/l tolbutamide (Fig. 3), a concentration that fully saturates the high-affinity-binding site for sulfonylureas. There was no difference in the potency of block for wild-type and mutant channels: 91 ± 3% (n = 4) *v *84 ± 7% (n = 4) respectively (Fig. 3).

### The patient and first-degree relatives

The patient with the *ABCC8 *mutation was 13 years old when diagnosed (Table 2). He had a high risk HLA profile (DR 04/04) and became positive for IA-2A 6 months after diagnosis. He had no first-degree relatives with diabetes. The mother was negative for the mutation in the *ABCC8 *gene and the father was unavailable for mutation analysis. Although functional analyses showed that the mutant channel was highly sensitive to sulfonylureas, there was no clinical effect on metabolic control or insulin requirement after four weeks of glibenclamide treatment (1.0-1.2 mg/kg/24h) 8 years after diagnosis of diabetes. We believe that the patient developed T1 D in addition to the *ABCC8*-diabetes as he now is insulin-dependent, C-peptide- and IA-2A-negative but GADA-positive (14.9 U/ml, cut-off limit is 10 U/ml) and therefore had no beneficial effects of sulphonylurea treatment. Meal-stimulated GLP-1 and GIP did not differ between the subject carrying the R1530C mutation and non-carriers (data not shown).

## Discussion

This study shows that 9,1% of children and adolescents with newly diagnosed type 1 diabetes did not have autoantibodies for ICA, GADA and IA-2A on initial testing or on 12 months follow-up (Table 1). This is consistent with a recent study on children and adolescents by Rubio-Cabezas where 9,9% tested negative for the same pancreatic autoantibodies(20)

We find that, when investigated in a physiologic setting, residual beta-cell function was considerably improved in autoantibody-negative (GADA, ICA, IA-2A) children with T1 D compared with autoantibody-positive (GADA, ICA, IA-2A) T1 D one year after diagnosis (Fig. 1A,B). The patients also had better glycemic control and required less exogenous insulin underscoring the milder disease process in patients with autoantibody-negative T1 D (Fig. 1C,D,E). We measured autoantibodies at 1, 6, and 12 months after diagnosis, and since we had no measurement at diagnosis, theoretically we cannot rule out the possibility that some patients might have been autoantibody positive at diagnosis and during the first month experienced seroconversion to antibody-negativity. On the other hand, none of the 239 subjects who were autoantibody-positive at one month converted to autoantibody-negative within the 12 months after diagnosis. We decided to exclude IA from our definition of autoantibody negativity, because we did not measure autoantibodies at onset. We do not think this biased our study, as at disease presentation very few patients are positive for insulin autoantibodies only (21). However, in the present study autoantibody-negative patients also had lower titers of IA at 1 month after diagnosis, further underscoring a lower immunological response and milder disease progression in this group of patients.

Recently it has been established that presence of autoantibodies directed against the beta cell zinc transporter ZnT8 are associated with type 1 diabetes. We have not included ZnT8 antibodies in our study but in a recent study in adults 1.4% of the GADA and IA-2A negative patients were ZNT8 positive (22). Therefore in future studies the inclusion of ZnT8 AB is mandatory to differentiate clinical phenotypes.

In our study we did not find any association with antibody (ICA, GADA, IA-2A) status and HLA risk alleles. It is possible that this could be explained by the absence of ZnT8 antibody status in the present study, and that inclusion of these might have revealed such an association.

Our findings indicate that a positive family history of diabetes may play a role, not related to the classical T1 D loci (HLA and *INS-VNTR)*. An important question is whether the 22 patients who remained autoantibody-negative truly had T1 D. At study entry the diagnosis of diabetes mellitus was made according to the WHO recommendations, and the responsible clinician at each center established the diagnosis of T1 D by her/his clinical judgment. Patients with suspected type 2 diabetes and patients with a family history of diabetes in three consecutive generations and with onset before the age of 25 years (suspected monogenic diabetes) were excluded. In the 6 antibody-negative patients with a positive family history, we did not find any mutations in *HNF1A *or *HNF4A*. We did not screen patients without a family history of diabetes for mutations and do not think we overlooked monogenic diabetes in these patients as spontaneous mutations are rare, although not impossible, in the transcription factor MODY genes (15, 17, 23, 24). On the other hand, neonatal diabetes due to mutations in *INS *(4, 17, 25) or either of the genes encoding the K-ATP subunits KIR6.2 (5) and SUR1 (6, 7) occurs *de novo *in approximately 50% of the cases and these subjects typically present with autoantibody-negative insulin-dependent diabetes. Relevant for the present study is our recent observation that *INS *mutations can be a cause of antibody-negative diabetes presenting as T1 D (18). Thus, we regarded *INS*, *KCNJ11 *and *ABCC8 *being good candidate genes in our antibody-negative patients.

We did not find pathogenic mutations in *INS *or *KCNJ11*, but one subject had a heterozygous mutation (R1530C) in SUR1 encoded by *ABCC8*. We believe this mutation is pathogenic because: (1) arginine at codon 1530 is conserved through evolution from zebrafish to humans; (2) codon 1530 is located in the second nucleotide-binding domain of SUR1, and other mutations in this domain cause permanent and transient neonatal diabetes and (3) functional analysis showed that homozygous mutant channels were ~three-fold more active at rest than wild-type channels. This suggests that the K-ATP current magnitude will also be increased in the beta-cell and in the heterozygous state, and can explain the diabetes of the patient.

Although our functional analyses showed that the channel was highly sensitive to sulphonylureas, our patient (8 years after diagnosis of diabetes) did not benefit from glibenclamide after 4 weeks of treatment (1.0-1.2 mg/kg/24h) in terms of metabolic control and insulin requirement. The 13-year-old male patient seroconverted to IA-2A positivity 6 months after diagnosis. It has been shown that adult-onset diabetes caused by SUR1 mutations responds favorably to sulphonylurea treatment (26). A possible explanation for the unsuccessful sulphonylurea treatment in our patient, despite promising functional studies of the K-ATP-channel, might be that the patient developed autoimmune diabetes. He had a high risk HLA profile, which might also facilitate the progression to autoimmune diabetes.

## Conclusions

In conclusion GADA, IA-2A, and ICA-negative children with T1 D have slower disease progression including better preservation of β-cell function and improved glycaemic control 12 months after diagnosis. A mutation within the *ABCC8 *gene may be a, so far, unidentified cause of autoantibody-negative childhood-onset diabetes, also after the neonatal period.

## Abbreviations

BMI: body mass index; GADA: glutamic acid decarboxylase autoantibodies; IAA: insulin autoantibodies; IA-2A: insulinoma-associated antigen-2 autoantibodies; ICA: islet cell antibodies; K-ATP channel: ATP-sensitive potassium channel; Monogenic Diabetes: maturity-onset diabetes of the young; RU: relative units.

## Competing interests

The authors declare that they have no competing interests.

## Authors' contributions

SP coordinated and contributed to the design of the studies, contributed to the interpretation of the results and wrote the manuscript. HBM; LH, PRN, ES, JÅ, PS; MK; EJS; CB; AM, designed the study, were responsible for patient enrolment and contributed to the interpretation of the results and the writing and the critical review of the manuscript. LN, MLMA carried out molecular genetic studies on the INS-VNTR and contributed to the interpretation of the results and the critical review of the manuscript. LBL, TS, PRN carried out molecular genetic studies on SUR 1 and MODY genes and contributed to the interpretation of the results and the writing and the critical review of the manuscript. HW, FA carried out the functional studies and contributed to the writing and the critical review of the manuscript. PH performed the statistical analyses of the study and contributed to the interpretation of the results and the critical review of the manuscript. MKN carried out antibody assays and contributed to the interpretation of the results and the critical review of the manuscript. All authors read and approved the final manuscript.

Members of the Hvidøre Study Group on Childhood Diabetes who have contributed to the Remission Phase Study:

Henk-Jan Aanstoot, MD, Ph.D., Center for Pediatric and Adolescent Diabetes Care and Research,,Rotterdam, The Netherlands; Carine de Beaufort, MD, Clinique Pédiatrique,Luxembourg; Francesco Chiarelli, Professor MD, Clinica Pediatrica, Chieti, Italy; Knut Dahl-Jørgensen, Professor, MD, Dr Med. SCI and Hilde Bjørndalen Göthner, MD, Ullevål University Hospital, Department of Paediatrics, Oslo, Norge; Thomas Danne, Proffessor, MD, Kinderkrankenhaus auf der Bult, Hannover, Germany; Patrick Garandeau, MD, Unité D'endocrinologie Diabetologie Infantile, Institut Saint Pierre, France; Stephen A. Greene, MD, DC, University of Dundee, Scotland; Hilary Hoey, Professor, MD, FRCPI, University of Dublin, National Children's Hospital, Tallaght, Ireland; Reinhard W. Holl, Professor MD, University of Ulm, Germany; Mirjana Kocova, Professor, MD, Pediatric Clinic-Skopje, Republic of Macedonia; Pedro Martul, Professor MD, Ph.D, Endocrinologia Pediatrica Hospital De Cruces, Spain; Nobuo Matsuura, Professor, MD, Kitasato University School of Medicine, Japan; Henrik B. Mortensen, Professor, MD, Dr Med. SCI, Department of Pediatrics, Glostrup Hospital & University of Copenhagen, Denmark; Kenneth J. Robertson, MD, Royal Hospital for Sick Children, Yorkhill, Glasgow, Scotland; Eugen J. Schoenle, Professor, MD, University Children's Hospital, Zurich, Switzerland; Peter Swift, MD, Leicester Royal Infirmary Childrens Hospital, Leicester, UK; Rosa Maria Tsou, MD/Professor Manuel Fontoura, Paediatric Department Oporto, Portugal; Maurizio Vanelli, Professor, MD, Clinica Pediatrica, Centro di Diabetologia, University of Parma; Jan Åman, MD, Ph.D, Örebro Medical Centre Hospital, Department of Paediatrics, Sweden

## Pre-publication history

The pre-publication history for this paper can be accessed here:

http://www.biomedcentral.com/1472-6823/10/16/prepub
